# Differential Population Responses to White‐Nose Syndrome Between Two Michigan Bat Hibernacula Are Not due to Differences in Host Susceptibility

**DOI:** 10.1002/ece3.72373

**Published:** 2025-12-17

**Authors:** Travis McDevitt‐Galles, Lisa E. Powers, Allen Kurta, Carol Meteyer, Tonie E. Rocke

**Affiliations:** ^1^ U.S. Geological Survey National Wildlife Health Center Madison Wisconsin USA; ^2^ Department of Biology Eastern Michigan University Ypsilanti Michigan USA

**Keywords:** *Myotis*, population effect, *Pseudogymnoascus destructans*, survival analysis, white‐nose syndrome

## Abstract

Disease outcomes result from the interaction between host, pathogen, and environmental factors. Understanding how these components interact to influence spatial and temporal variations in disease severity can enhance our insights into the drivers of disease outbreaks, ultimately improving our ability to mitigate the impact of disease through better forecasts and management actions. White‐nose syndrome (WNS) in bats, caused by the fungal pathogen *Pseudogymnoascus destructans* (Pd), has been detected in hibernating bats across much of the United States and Canada. This pathogen has led to widespread population declines in some bat species, for example, *Myotis lucifugus* ; however, not all infected populations exhibit similar decreases in numbers. Despite long‐term detection and high infection levels, the population of *M. lucifugus* that uses Tippy Dam, in northern Michigan, as a hibernaculum has not experienced a decline compared to other populations in the state. To assess local population effects that may contribute to reduced disease severity at Tippy Dam, we brought 30 hibernating *M. lucifugus* from Tippy Dam and 30 from a geographically similar hibernaculum with a history of declines from WNS into captivity at the U.S. Geological Survey, National Wildlife Health Center. We challenged the bats with a Pd inoculum and monitored survival, pathology, and Pd loads for up to 120 days. This allowed us to remove local environmental effects that could influence WNS disease severity. We observed no effect of source population on either survival or wing damage from Pd infection. Our results suggest that population persistence and lowered disease severity in Tippy Dam are likely driven by local environmental factors found within the dam. As Pd continues to spread westward, understanding environmental factors that influence the severity of Pd infection in hibernating bats has the potential to guide management decisions and help predict the survival of susceptible bat species in the western United States.

## Graphical abstract

The study investigates the factors influencing the reduced severity of white‐nose syndrome (WNS) in *Myotis lucifugus* bats at Tippy Dam, Michigan, despite high infection levels. Through controlled experiments, we show that environmental factors, rather than host adaptation, play a critical role in the bats' long‐term persistence and lower disease severity.

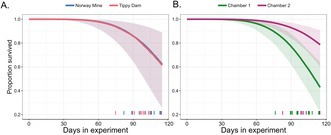

## 1 Introduction

The spread of a novel pathogen across a landscape can have devastating impacts on wildlife populations (Daszak et al. 2000; Harvell et al. 2002; Fisher et al. 2012), though variable outcomes may occur across the host population's range (Paull et al. 2012; Stephens et al. 2016). Certain populations may experience severe declines and local extirpations (McCallum 2012), whereas other populations may have limited or no noticeable effects, despite having a similar level of pathogen exposure or infection (Wilber et al. 2017). For example, the global spread of the amphibian chytrid fungus, *Batrachochytrium dendrobatidis*, has reduced amphibian populations, leading to extinction and extirpation events, but populations and communities of some amphibian species persist even under intense infection pressure (Scheele et al. 2019; Fisher and Garner 2020). Population persistence in this system can be driven by both intrinsic (i.e., immunity, genetics, and behavior) and extrinsic (i.e., density dependence, temperature, and salinity) factors (Bacigalupe et al. 2017; Brannelly et al. 2021). This insight into population persistence has allowed researchers to develop evidence‐based mitigation guidelines to help improve host outcomes during the invasion and spread of *B. dendrobatidis* (Woodhams et al. 2011; Scheele et al. 2014). Thus, understanding specific mechanisms that drive host persistence can provide management tools to mitigate the effects of disease on wildlife populations.

White‐nose syndrome (WNS), a bat disease caused by the cold‐adapted fungus, *Pseudogymnoascus destructans* (Pd), has led to significant population declines in several North American species since its detection in eastern North America in 2007 (Blehert et al. 2009; Cheng et al. 2021; Hoyt et al. 2021). *Pseudogymnoascus destructans* colonizes the skin of hibernating bats, disrupting physiological processes that deplete energy resources and often result in death (Cryan et al. 2010; Verant et al. 2014). The fungus is well established across the midwestern and eastern United States and Canada, and it continues to expand its geographic range westward (Lorch et al. 2013, 2016; Wiens and Thogmartin 2022). Understanding the factors—whether extrinsic (environmental) or intrinsic (host traits)—that promote population persistence in the face of ongoing WNS threats in eastern North America (Cheng et al. 2021) could enhance our ability to manage western populations before, during, and after Pd invades a colony. For instance, if limited declines during the initial Pd invasion are largely driven by extrinsic factors, such as local environmental characteristics, managers can use this information to identify environmentally similar sites that may offer natural protection and allow allocation of resources to more vulnerable locations. Conversely, if persistence is attributed to intrinsic factors, such as variation in fat deposition (Cheng et al. 2019), managers could allocate resources to promote favorable conditions for the identified intrinsic factors in western colonies, such as increased food availability.

Unlike most hibernacula in eastern North America, no mass mortality events have been observed in little brown bats ( *Myotis lucifugus* ) hibernating in the spillway at Tippy Dam, a hydroelectric facility in northern Lower Michigan, despite the presence of Pd on the walls and on the bats there since the winter of 2014–2015 (Kurta et al. 2020). This population of hibernating *M. lucifugus* has remained steady at 20,000–25,000 individuals throughout the course of Pd introduction, invasion, and establishment (Gmutza et al. 2024). The long‐term population stability at Tippy Dam makes it a unique site to investigate factors that could influence the persistence and stability of *M. lucifugus* despite the occurrence of Pd. This hibernaculum is unusual in several respects; for example, dim light filters through numerous openings in the spillway, and internal temperatures fluctuate considerably on an annual basis (from about 2°C to 24°C; Daly 2024; Kurta et al. 1997; Gmutza et al. 2024, Allen Kurta, unpublished data). In addition, the internal environment is subjected to constant low‐frequency sound (< 150 Hz), and the structure itself vibrates continually at 120.12 Hz (Rodney W. Foster, unpubl. data). Localized intrinsic factors, such as host behavioral and physiological traits, could also promote bat colony persistence through beneficial microhabitat selection or strong innate immune response to infection (Auteri and Knowles 2020). Disentangling the drivers of host persistence at Tippy Dam could further illuminate the primary factors that contribute to population resilience in the face of WNS.

To assess whether the survival of bats using Tippy Dam for hibernation is due to intrinsic population traits or a possible environmental factor, we conducted a comparative survival experiment with *M. lucifugus* from Tippy Dam and from a geographically similar hibernaculum that had documented population declines from WNS in preceding years. By removing the bats from their hibernaculum, challenging them with the same source and quantity of Pd, and placing them in shared environmental chambers, we removed the influence of local environmental conditions. Here, we report comparisons of survival, pathologic changes in wing tissues, and Pd growth between bat populations.

## 2 Methods

### 2.1 Source Population Sites

We compared bat survival and other dynamics related to WNS within controlled environments between two distinct populations of *M. lucifugus* from hibernating colonies in Michigan. Our focal colony is located in Tippy Dam, a hydroelectric facility on the Manistee River near Wellston, Manistee County, Michigan, where *M. lucifugus* hibernate in the hollow spillway of the dam. In comparison, *M. lucifugus* colonies in the Upper Peninsula of Michigan that use abandoned copper and iron mines have experienced an overall decline of 89.9% since the introduction of Pd (Kurta and Smith 2020). One of these mines, the Norway Mine, located near Norway, Dickinson County, Michigan, had a large decline of hibernating bats, from 17,000–24,000 *M. lucifugus* pre‐WNS to a roosting population of only 3000 in 2022. Norway Mine is a disused iron mine with a steeply sloping entrance and multiple chambers where bats hibernate.

### 2.2 Experimental Design

Thirty hibernating male *M. lucifugus* were collected from both Tippy Dam and Norway Mine (for a total of 60 hibernating bats) on 15 and 16 December 2022, respectively, when the ambient temperature inside both hibernacula was 6°C–7°C. Bats were immediately transported to the National Wildlife Health Center (NWHC) in Madison, Wisconsin. Upon arrival, bats were weighed, sampled for initial Pd loads, and given unique wing bands following standard protocol (refer to Rocke et al. 2019). We placed bats in two hibernation chambers that each contained two cages 15 cm apart; half of the bats from one population (Tippy Dam or Norway Mine) were placed in one cage in each chamber. The chambers were held at a stable temperature (6°C–10°C) and relative humidity (85%–95%). After acclimating for 14 days, all bats were given a 20 μL dose of freshly prepared Pd inoculum at a concentration of 2.5 × 10^4^ conidia/μL directly on their wings, following the protocol developed and described in detail in Rocke et al. (2019). Bats were observed twice daily for up to 120 days postexposure to detect sick or dead bats. On days 30, 60, and 90, surviving bats were swabbed to quantify changes in Pd loads, whereas dead bats were removed and necropsied. Bats that survived the experiment were euthanized, necropsied, and sampled for Pd loads at ~day 120. Wing tissues were collected from each for histological examination.

To determine Pd load, we swabbed the length of the dorsal side of wing membranes three times per side for each bat with a pre‐wetted, sterile cotton swab. We stored the swabs in reagent‐grade sterile water at −20°C before quantifying Pd load through quantitative polymerase chain reaction (qPCR) (Muller et al. 2013). Upon necropsy of bats found dead or euthanized, we cut the plagiopatagium tissue from the right wing, prepared it for paraffin processing, and stored it in 10% neutral buffered formalin following the methods previously described (Meteyer et al. 2009). Histologic changes in the wings were scored on the basis of WNS lesion severity and were given a value between 0 and 4. In brief, the severity scores are based on both the degree and extent of erosions on the wing because of the Pd fungal hyphae, with a score of 0 for unaffected individuals and a score of 4 for severely affected. A full description of the histologic scoring methods can be found in appendix S2 of (Reeder et al. 2012).

### 2.3 Statistical Approach

Our primary goal was to determine whether the source population (Tippy Dam or Norway Mine) affected Pd infection of bats and its outcome. To be comprehensive, we examined three distinct components of bat‐Pd dynamics: (1) host survival, (2) wing pathology, and (3) Pd loads on wings over time. We developed three separate models for each component, which we describe in detail below. We conducted data cleaning and visualizations using the R statistical language (R Core Team 2023) and various tidyverse packages (Wickham et al. 2019), including ggplot2 (Wickham and Wickham 2016) and dplyr (Wickham 2014). We constructed all models within a Bayesian framework using the Stan language (Carpenter et al. 2017) and fit in R using both the rstan and brms (Bürkner 2017) packages. We assessed model convergence by inspecting the Gelman–Rubin test statistic, with a value < 1.2 indicating convergence (Gelman and Rubin 1992). Additionally, we visually inspected the trace plots to ensure proper mixing and the absence of divergent transitions. For all models, we ran four chains with 2000 iterations, with a 1000 iteration burn‐in period.

#### 2.3.1 Survival Model

To assess if the source population influenced the survival of bats exposed to Pd, we developed a parametric Weibull survival model (Landes et al. 2020). The model assumes that the time to event (death or censoring) follows a Weibull distribution defined by a shape (*α*) and a scale parameter (*τ*). The *α* parameter allows us to account for when a bat is most likely to die. If *α* < 1, then a bat is more likely to die early in the experiment, and if *α* > 1, then a bat is more likely to die later in the experiment. If *α* = 1, then the Weibull distribution reduces to the exponential distribution. We use the scale parameter τ to define our hazard model and include covariates to assess how various factors influence survival. We define *τ* following Peltola et al. (2014).

$$\tau =e^{-\left(\frac{\left(\mu +\beta *X\right)}{\alpha }\right)}.$$

The parameter *μ* or the intercept is baseline survival, and parameter *β* is the estimated coefficients of the survival covariates *X*, which describe how each covariate impacts survival. The estimated survival coefficients capture the effect of the given covariate on when the event (in our case, death) would occur. Positive values indicate quicker times to the event occurring (higher risk of mortality or lower survival), whereas negative values indicate slower times to the event (lower risk of mortality or higher survival). The parameter *α* is the same shape parameter mentioned above. For our covariates, we included source population (Tippy Dam or Norway Mine), chamber (chamber one or two), and initial weight in grams. We included initial weight because starting weight is an important predictor of bat survival during hibernation (Kunz et al. 1998). For both the *α* and *β* parameters, we used a normal prior centered on zero with a standard deviation of three. For the *μ* or intercept parameters, we used a normal prior centered on zero with a larger standard deviation of five to allow for possibilities of larger values.

#### 2.3.2 Wing Pathology Model

As our histology data are defined along a rank‐ordered categorical scale (i.e., WNS severity score of 0–4), we used an ordinal regression model to explore factors that shaped WNS severity during our experiment. We used a cumulative ordinal model as it assumes our categorical scale (WNS severity score) is estimated from a latent continuous variable (wing pathology from Pd infection) (Bürkner and Vuorre 2019). For our predictor variables, we used source population, chamber, and Pd loads on the natural log scale with a probit‐link function. To improve model fit, we z‐transformed our Pd load data. For all coefficients, we used standard normal priors.

#### 2.3.3 Pd Load Model

We modeled changes in log_10_‐transformed Pd loads across time, using a linear mixed‐effects model with a Gaussian distribution. For our predictor variables, we used source population, chamber, and day since Pd challenge (time). Since we were primarily focused on changes in Pd loads across time, we included interactions with time and both source population and chamber, as well as a three‐way interaction among time, source population, and chamber. As our data represent resampling of individuals across time, we used a random intercept term for bat identifier. For all coefficients, we used standard normal priors.

## 3 Results

In total, we exposed 60 male *M. lucifugus* bats to a standardized dose of Pd. Of the 60 bats, 60% (36/60) survived until the end of the experiment (Table 1 in Appendix S1A). The mean time to death for bats that did not survive (24 bats) was 100 days (range 76–114 days) (Appendix S1B). At the start of our experiment, bats from Tippy Dam had a mean weight of 7.49 g (0.42 SD), whereas bats from Norway Mine had a mean weight of 7.79 g (0.73 SD) (Appendix S1C). We obtained WNS severity scores for 59 of 60 individuals by histologic analysis (Table 2 in Appendix S1D); one wing sample was inadvertently omitted during sample collection. Over half (54%) of the sampled bats had a low WNS wing score of either 0 or 1 (32/59), 39% had a moderate score of 2 (23/59), and 7% had a high score of 3 or 4 (4/59).

### 3.1 Survival Model

**FIGURE 1:**
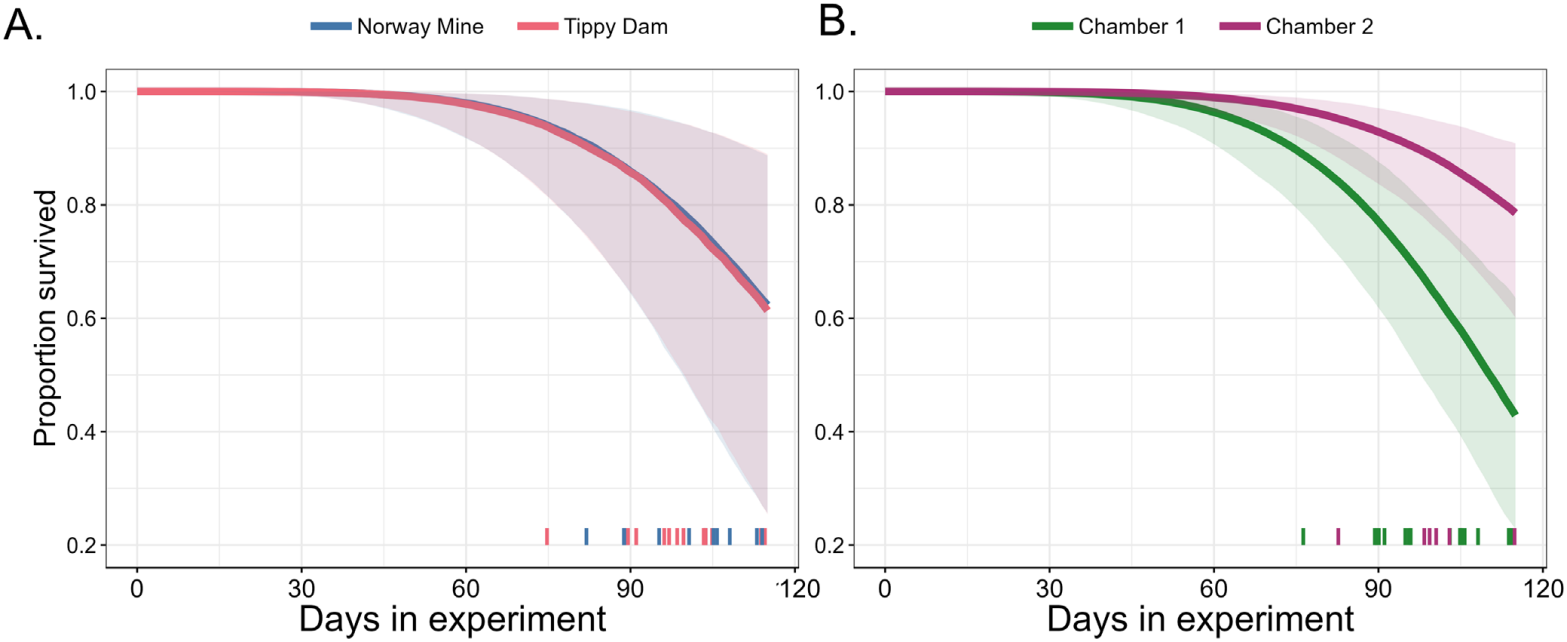
Model outputs of the survival analysis for the *Pseudogymnoascus destructans* 120 day exposure experiment in *Myotis lucifugus* bats. (A) Source population of the hibernating bats (blue for bats from Norway Mine, MI and red for Tippy Dam, MI) did not have an impact on survival. Solid lines show the median estimated proportion surviving throughout the duration of our experiment and shaded areas represent the 95% credible intervals (CI). Because the median parameter estimation centered on 0 (Estimated parameter with 95% CI: 0.04 [−0.68, 0.76]), we observed strong overlap in estimated survival across time for both populations. Solid ticks along *x* axis indicate times of death for bats from both populations with a small jittering along the *x*‐axis to improve visualization of overlapping times of death. (B) The chamber in which bats were housed affected their survival (estimated parameter with 95% CI of Chamber two −1.30 [−2.34, −0.97]), with bats in Chamber two (purple) having higher survival than bats in Chamber one (green). Solid lines indicate the median predicted values of survival across time, and the shaded areas show the 95% credible intervals. Solid ticks along the *x*‐axis indicate times of death for bats from either chamber.

**FIGURE 2:**
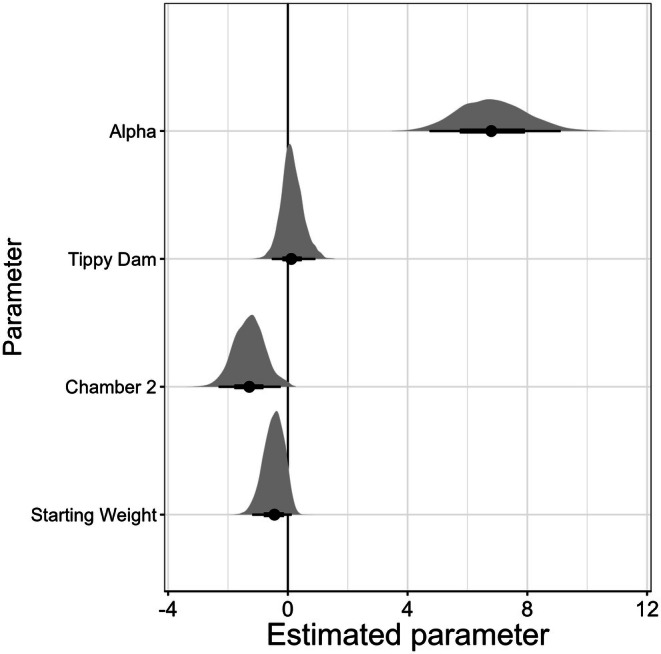
The posterior distribution of parameters of interest from our Weibull survival model for hibernating *Myotis lucifugus* bats from two hibernacula in Michigan, Tippy Dam and Norway Mine, after exposure to the fungal pathogen Pd for 120 days. Positive values indicate higher associated mortality for the given parameter, and negative values indicate lower mortality. Circles indicate the median parameter estimation, with the black bars showing the 95% distribution of the estimated parameter value. The gray fill shows the full distribution of the parameter estimation.

For our survival model, we evaluated both the Weibull shape parameter *α* and how our experimental covariates impacted survival through the scale parameter *τ*. On the basis of our estimated *α* parameter (mean estimated coefficient with 95% credible intervals [CI]: 6.83 [4.74, 9.11]), the bats in our study had high survival early in the experiment, with deaths occurring only during the later end of the experiment (Figure 1). Overall, our *μ* or intercept parameter was −28.94 [−39.67, −18.59], indicating high survival for our baseline population (bats from Norway Mine in Chamber one). Bats from Tippy Dam had no difference in survival compared to the reference Norway Mine bats (mean estimated Tippy Dam *β* with 95% CI: 0.14 [−0.53, 0.92]) after accounting for chamber and initial weights (Figure 1A). Bats housed in Chamber two survived longer compared to bats housed in Chamber one (mean estimated Chamber two *β* with 95% CI: −1.29 [−2.31, −0.24], Figure 1B). Bats that had higher initial weights had longer survival times (mean estimated weight *β* with 95% CI: −0.47 [−1.19, 0.13], Figure 2), though the tail of the distribution does slightly overlap with 0. The model diagnostics indicated full mixing of our chains and model convergence, with a maximum Gelman–Rubin test statistic of 1.01.

### 3.2 Wing Pathology Model

**FIGURE 3:**
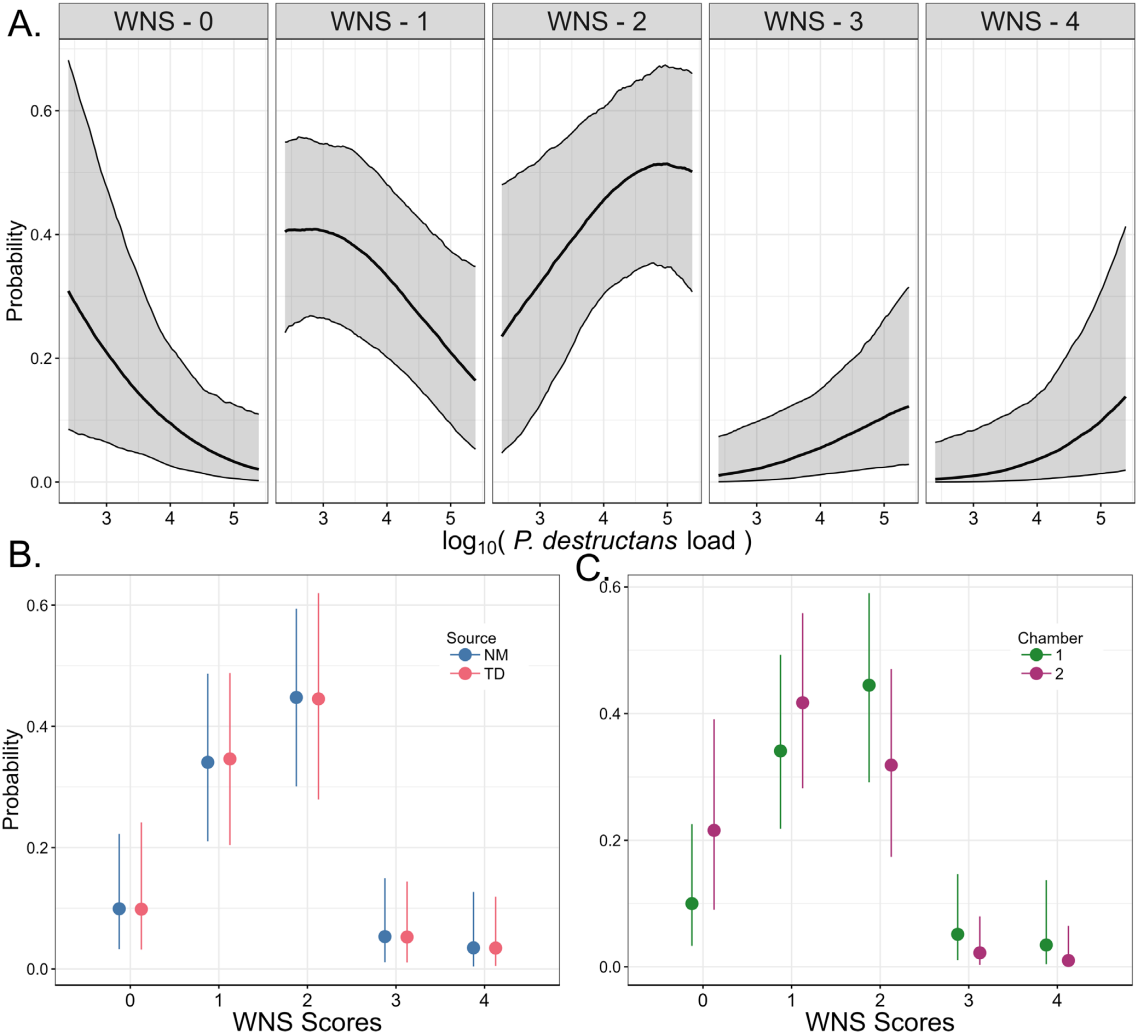
White‐nose syndrome (WNS) severity scores on hibernating *Myotis lucifugus* bats were driven by both infection load of the causal agent (*Pseudogymnoascus destructans*) and the chamber in which the bats were housed, but the source population had no impact on WNS severity score probability. (A) Increase in the probability of a bat receiving a higher WNS severity score (scores ranged from 0 to 4) was largely driven by infection load of *P. destructans* (estimated coefficient with 95% credible interval (CI): 0.38 [0.07, 0.69]). Bats with lower infection loads tended to have a lower score of 0 or 1, whereas bats with higher infection loads tended to have a higher score of 3 or 4. Solid black lines show the median predicted relationship between infection load and probability of being assigned a given WNS severity score, and gray fill area indicates 95% CI. Each box represents the relationship between infection load and probability for each WNS severity score (0–4). (B) Bat source population had no detected effect on WNS severity score (estimated Tippy Dam, MI coefficient with 95% CI: 0.08 [−0.49, 0.64]). Bats from Tippy Dam (TD, blue) had similar WNS severity scores compared to bats from Norway Mine, MI (NM, red). Each point indicates the median predicted probability of the associated WNS severity score on the basis of chamber, with the vertical lines indicating the 95% CI. (C) Bats housed in Chamber two (purple) had a lower chance of having higher scores compared to bats housed in Chamber one (green) (Estimated Chamber two coefficient with 95% CI: −0.46 [−1.05, 0.11]). Each point indicates the median predicted probability of the associated WNS severity score on the basis of chamber, with the vertical lines indicating the 95% CI.

The ordinal model of wing pathology identified several factors impacting the probability of an assigned WNS severity score. Bats with higher Pd loads at death or at the end of the experiment were more likely to have higher WNS severity scores, indicating more severe disease (mean estimated Pd load *β* with 95% CI: 0.36 [0.07, 0.66]) than bats with lower initial Pd loads (Figure 3A). Similar to our survival model, we observed no effect of source population on WNS severity score probability (mean estimated Tippy Dam *β* with 95% CI: 0.00 [−0.54, 0.56], Figure 3B). A chamber effect on WNS severity score probability was observed with bats housed in Chamber two having slightly lower severity scores (mean estimated Chamber two *β* with 95% CI: −0.49 [−1.03, 0.05], Figure 3C), though the 95% CI does overlap 0. The model diagnostics indicated full mixing of the chains and model convergence with a maximum Gelman–Rubin test statistic of 1.00.

### 3.3 Pd Load Model

**FIGURE 4:**
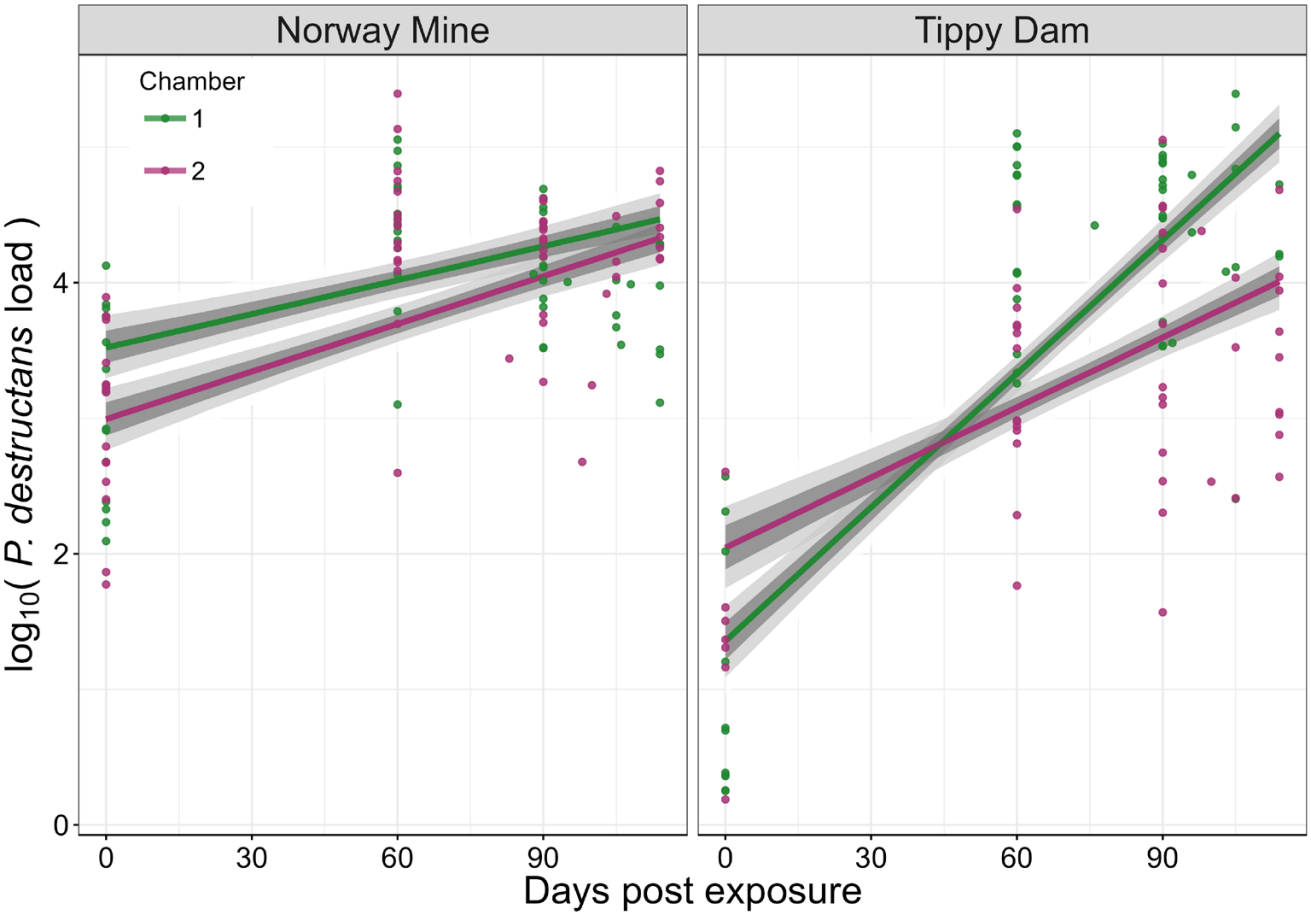
Patterns of *Pseudogymnoascus destructans* (Pd) load on *Myotis lucifugus* bat wings varied across time, source population, and the chamber the bats were housed in over the course of the 120 day exposure to Pd. At the onset of the experiment (day 0, before Pd challenge at the U.S. Geological Survey's National Wildlife Health Center (NWHC)), we observed lower Pd loads (fg, log10 transformed) in hibernating bats from Tippy Dam, MI compared to hibernating bats from Norway Mine, MI (estimated coefficient with 95% credible interval (CI): −0.59 [−0.85, −0.34]). For both populations, Pd loads increased across time, with higher rates of increase in the bats from Tippy Dam (estimated time coefficient with 95% credible interval (CI): 0.34 [0.15, 0.52], estimated time X Tippy Dam interaction coefficient with 95% credible interval (CI): 1.00 [0.71, 1.28]). However, Tippy Dam bats housed in Chamber two had lower rates of increase compared to bats in Chamber one (estimated time X Tippy Dam X Chamber two interaction coefficient with 95% credible interval (CI): −0.77 [−1.18, −0.35]). Each dot represents Pd infection load for an individual time point, with green dots representing bats in Chamber one and purple from Chamber two. Solid lines are the median predicted relationship between days post exposure and Pd load for chamber and source identity. The shaded lines indicate the 50% and 95% CI.

Bats sourced from Tippy Dam had lower initial Pd loads (mean log_10_ Pd loads in Femtograms ±1 SD: 1.16 ± 0.82, Appendix S1E) at capture in early December (mean estimated Tippy Dam *β* with 95% CI: −0.59 [−0.85, −0.33]) compared to bats captured at Norway Mine at the same time (mean log_10_ Pd loads ±1 SD: 3.03 ± 0.64, in Appendix S1E). After the Pd challenge at NWHC, loads increased across time for all bats (mean estimated scaled day *β* with 95% CI: 0.34 [0.16, 0.52]). Pd loads on Tippy Dam bats increased at a higher rate compared to Norway Mine bats in Chamber one (mean estimated Tippy Dam—Chamber one scaled day three‐way interaction *β* with 95% CI: −0.78 [−1.18, −0.38], Figure 4), but not in Chamber two. The model diagnostics indicated full mixing of our chains and model convergence with a maximum Gelman‐Rubin test statistic of 1.00.

## 4 Discussion

Tippy Dam is notable for its low mortality in bats affected by WNS, relative to typical hibernacula throughout the eastern United States and Canada (Kurta et al. 2020), which has led to questions of whether this observation is due to environmental factors or bat population traits. In our captive challenge experiment, *M. lucifugus* from Tippy Dam and Norway Mine had similar survival and wing pathology after exposure to Pd in shared hibernation chambers. The most notable difference between the two groups was that bats from Tippy Dam had significantly lower Pd loads upon intake in early December compared to bats from Norway Mine, though after challenge, Pd loads on bats from Tippy Dam increased at a faster rate than loads on bats from Norway Mine. These combined results indicate that local environmental conditions are more likely related to lower WNS‐related mortality at Tippy Dam than host factors, such as differences in initial fat reserves or differences in Pd susceptibility.

Fungal load in the early hibernation period correlated with the impact of WNS in several bat species at other locations (Langwig et al. 2016; Frick et al. 2017). Numerous species of bats (including *M. lucifugus* ) with lower Pd loads during early hibernation generally experience lower mortality from WNS over winter than those with higher loads (Langwig et al. 2016). The Pd loads on *M. lucifugus* detected upon capture at Tippy Dam during early hibernation were similar to published Pd loads of bat species known to be less impacted by WNS (e.g., *Eptesicus fuscus* ; Langwig et al. 2016). Lower fungal loads on bats during early hibernation could result from lower initial loads in the environment or environmental conditions that do not facilitate germination and growth of fungal spores on bats. For example, the concrete substrate in Tippy Dam may not favor retention of fungal spores in the environment between seasons (but see Fischer et al. 2020), or annual temperature fluctuations within the dam may affect fungal germination or bat activity during hibernation (Johnson et al. 2014; Urbina et al. 2021; Loeb et al. 2025). Lower fungal loads in early winter may not allow the fungus to reach levels that cause severe morbidity or mortality by the time the bats emerge from hibernation in spring. In a follow‐up survey in late winter (29 February–1 March) 2024, Pd loads on 30 bats sampled at each source location were significantly lower on Tippy Dam bats than Norway Mine bats (refer to Appendix S1F in Table 3). In addition, though unlikely, it cannot be ruled out that a reduction in Pd loads could be related to a unique strain of Pd found at Tippy Dam. The variation in pathogen virulence can play an important role in shaping the spatio‐temporal patterns of population persistence (Johnson et al. 2024). More work on bats in Tippy Dam could clarify patterns of Pd growth in this population.

In our challenge study, bats from both Tippy Dam and Norway Mine in Chamber 2 survived longer and at higher rates than bats from Chamber 1. During the course of the experiment, we observed increasing levels of green fluorescence over time on the bats' wings from both source populations in Chamber 2 that were rarely observed on bats in Chamber 1. Bacterial culture of swabs collected from bats with green fluorescence and subsequent genetic analysis revealed the presence of *Pseudomonas* spp., some of which have a protective effect for bats infected with Pd, whereas others have limited or negative effects (Rainey and Travisano 1998; Brucker et al. 2008; Mühldorfer 2013; Hoyt et al. 2015). We could not confirm which bats were the initial source of the microbe, so no further analyses were conducted. However, the presence of inhibitory microbes as an environmental factor that protects bats from WNS warrants further consideration.

Our findings suggest that environmental conditions specific to Tippy Dam result in lower WNS mortality rates than are typical for *M. lucifugus* in eastern North America. This aligns with the conclusions of Gmutza et al. (2024), who demonstrated that light in the dam results in synchronous arousal of bats. They hypothesized that facilitated social thermoregulation, leading to lower energy loss during arousals from hibernation, could protect bats from the premature depletion of fat reserves that characterizes WNS (Gmutza et al. 2024), although this mechanism has not been confirmed experimentally. Additional environmental factors at Tippy Dam, such as humidity (Langwig et al. 2012; Haase et al. 2021) and temperature (Verant et al. 2012; Wu et al. 2025), may also reduce the Pd burden and enhance bat survival. For example, warm temperatures inside the spillway in summer (≤ 24°C) may inhibit the survival of conidia, leading to a small environmental reservoir when the bats enter hibernation (Hoyt et al. 2021), and low temperatures in midwinter (≥ 2°C) may slow fungal growth (Verant et al. 2012). Furthermore, the growth of some ascomycete fungi is inhibited by exposure to low‐frequency sounds and vibrations, like those experienced in the spillway (Jeong et al. 2013; Razavizadeh et al. 2024), although it is unknown whether Pd is similarly affected. The floor of the 12‐m‐tall chambers within the spillway is constantly covered with shallow pools of water, although some additional water briefly enters on the rare occasions that the spill gates are opened. However, the water is physically restricted to the lower part of the chambers, where fewer than 1% of the bats roost, and it is not likely that this partial flushing of the bottom of the hibernaculum is having meaningful effects on Pd establishment and growth.

Although these environmental variables may mitigate Pd exposure, long‐term survival and population stability may also depend on intrinsic bat population traits. For instance, Grimaudo et al. (2022) conducted a translocation experiment and found that although Pd loads and WNS pathology were associated with warmer hibernacula temperatures and lower humidity, bats originating from warmer, drier climates utilized a broader range of microclimates compared to bats from colder, wetter areas. This behavior resulted in higher survival rates and lower Pd loads compared to bats from colder, wetter environments. These findings suggest that population stability likely arises from an interaction between favorable environmental conditions and adaptive population traits. In our study, we were not able to account for potential differences in microhabitat use or other population traits related to behavioral differences that might improve population stability. Further research could clarify the complexity of bat–environment interactions for long‐term stability during Pd invasion.

We suspect a combination of factors may be involved in bat persistence at Tippy Dam; further study could help pinpoint them. In addition, our study only included male bats, and as strong sex‐based differences are known to affect WNS dynamics (Grieneisen et al. 2015; Kailing et al. 2023), future studies on female bats are warranted as well. Quantifying the source of variation in bat population persistence or a better understanding of the primary factors associated with persistence could be used to improve predictions of WNS mortality as Pd becomes established in the western United States, where hibernacula environments differ from those in the East (Weller et al. 2018). Continued assessments of the impact of environmental conditions on bat mortality from WNS at western hibernacula would clarify the range of susceptibility in novel environments. These patterns of susceptibility can be used to help deploy various methods of mitigating WNS impacts in a strategic manner (e.g., anti‐fungal agents, biological controls, ultraviolet‐light treatment, vaccines; Gabriel et al. 2022; Hoyt et al. 2019, Kwait et al. 2022; Rocke et al. 2019). Wildlife managers could maximize the impact of efforts to protect bats from WNS if we are able to determine which hibernacula have environmental conditions that may prevent or lower WNS mortality and which may require treatment interventions.

## Author Contributions

**Travis McDevitt‐Galles:** conceptualization (equal), formal analysis (equal), visualization (equal), writing – original draft (equal), writing – review and editing (equal). **Lisa E. Powers:** conceptualization (equal), formal analysis (equal), writing – original draft (supporting), writing – review and editing (equal). **Allen Kurta:** conceptualization (equal), funding acquisition (equal), methodology (equal), project administration (equal), writing – review and editing (equal). **Carol Meteyer:** investigation (equal), methodology (equal), writing – review and editing (equal). **Tonie E. Rocke:** conceptualization (equal), data curation (equal), funding acquisition (equal), methodology (equal), project administration (equal), writing – review and editing (equal).

## Conflicts of Interest

The authors declare no conflicts of interest.

## Supporting information


**Appendix S1:** ece372373‐sup‐0001‐supinfo.pdf.

## Data Availability

Data are publicly available in a U.S. Geological Survey data release (McDevitt‐Galles et al. 2025).
