# Supplementary material for: Differential Population Responses to White‐Nose Syndrome Between Two Michigan Bat Hibernacula Are Not due to Differences in Host Susceptibility
Source: Ecol Evol. 2025 Dec 17;15(12):e72373. doi: 10.1002/ece3.72373 (PMC12711601; doi:10.1002/ece3.72373)
Supplement: Supplementary file 1 — Appendix S1: ece372373‐sup‐0001‐supinfo.pdf. [file ECE3-15-e72373-s001.pdf]

Supplementary Information for 'Differential population responses to White-nose syndrome between two Michigan bat hibernacula are not due to differences in host susceptibility'

Travis McDevitt-Galles, Lisa E. Powers, Allen Kurta, Carol Meteyer, and Tonie E. Rocke

Abstract

Disease outcomes result from the interaction between host, pathogen, and environmental factors. Understanding how these components interact to influence spatial and temporal variations in disease severity can enhance our insights into the drivers of disease outbreaks, ultimately improving our ability to mitigate the impact of disease through better forecasts and management actions. White-nose syndrome (WNS) in bats, caused by the fungal pathogen *Pseudogymnoascus destructans* (Pd), has been detected in hibernating bats across much of the United States and Canada. This pathogen has led to widespread population declines in some bat species, e.g. **Myotis lucifugus_; however, not all infected populations exhibit similar decreases in numbers. Despite long-term detection and high infection levels, the population of_ M. lucifugus* that uses Tippy Dam, in northern Michigan, as a hibernaculum has not experienced a decline compared to other populations in the state. To assess local population effects that may contribute to reduced disease severity at Tippy Dam, we brought 30 hibernating M. lucifugus from Tippy Dam and 30 from a geographically similar hibernaculum with a history of declines from WNS into captivity at the U.S. Geological Survey, National Wildlife Health Center. We challenged the bats with a single Pd inoculum, placed them in chambers and monitored survival, pathology, and Pd loads for up to 120 days. This allowed us to remove local environmental effects that could influence WNS disease severity. We observed no effect of source population on either survival or wing damage from Pd infection. Our results suggest that population persistence and lowered disease severity in Tippy Dam is likely driven by local environmental factors found within the dam. As Pd continues to spread westward, understanding environmental factors that influence the severity of Pd infection in hibernating bats has the potential to guide management decisions and help predict the survival of susceptible bat species in the western United States.

Document Overview

This document contains the supplementary materials for the manuscript titled "Differential population responses to White-nose syndrome between two Michigan bat hibernacula are not due to differences in host susceptibility"

- A) Summary table of survival experiment
- B) Histogram of times of deaths
- C) Density plot of initial and endpoint weights between the two source locations
- D) Table of individual bat White-Nose severity score
- E) Density plots of initial and endpoint Pd loads between the two source locations
- F) Field Pd load analysis

A) Summary table of survival experiment

Table 1: Hibernating bats (Myotis lucifugus) from hibernacula in Michigan (30 bats from Tippy Dam, 30 bats from Norway Mine) were exposed to the fungal pathogen Pseudogymnoascus destructans (Pd) for 120 days.

Source	Chamber	Total-bats	Total-deaths	Initial weight (grams)	End weight (grams)	Initial-Pd-Load	End-Pd-Load
Norway Mine	1	15	8	8.05	5.83	3.10	3.86
Tippy Dam	1	15	10	7.47	5.72	1.01	4.37
Norway Mine	2	15	3	7.53	5.74	2.96	4.10
Tippy Dam	2	15	3	7.52	6.09	1.39	3.44

B) Histogram of times of deaths

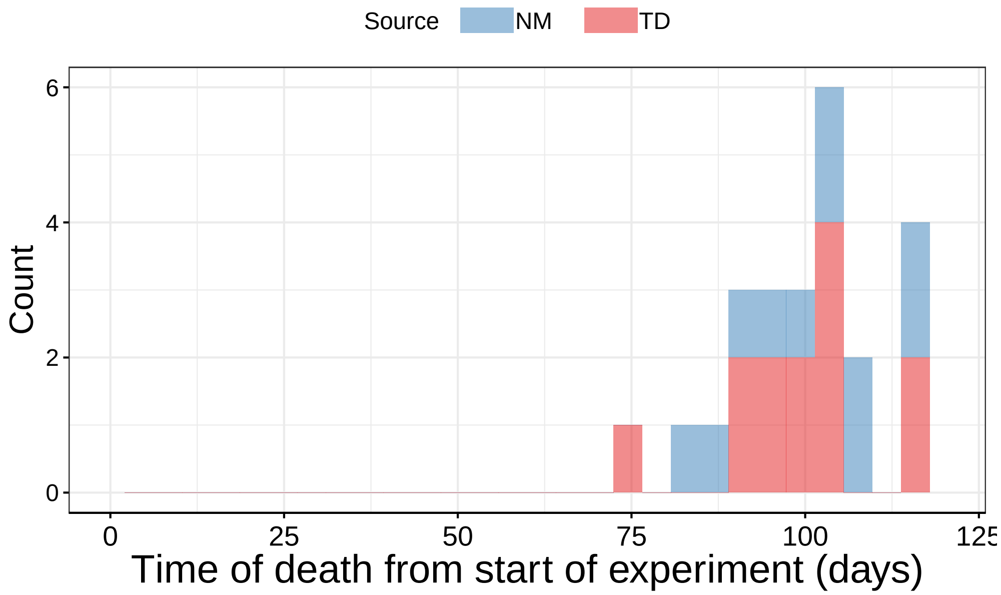

Figure 1: Distribution of time of death in our survival experiment. Hibernating bats (Myotis lucifugus) from hibernacula in Michigan (30 bats from Tippy Dam (TD), 30 bats from Norway Mine(NM)) were exposed to the fungal pathogen Pseudogymnoascus destructans (Pd) for 120 days. Red bars are time of deaths for tippy damTippy Dam bats and blue bars are for norway mineNorway Mine bats.

C) Density plot of initial and endpoint weights between the two source locations

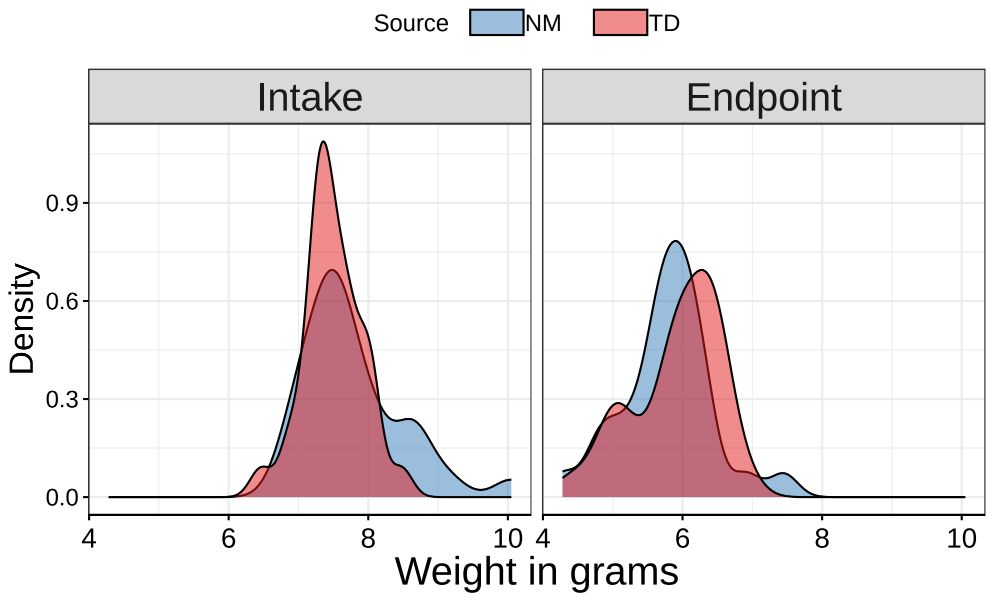

Figure 2: Density plot of weight of hibernating bats (Myotis lucifugus) in grams. The left plotplots show captures the weight distribution of bats from the two populations from hibernacula in Michigan (red for Tippy Dam (TD) and blue for Norway Mine (NM)) at intake (start of the experiment), and at endpoint the right plot is the distribution of weights at (the end of the experiment).

D) Table of individual bat White-Nose severity score

Table 2: Histology results

BatID	Chamber	Source	End weight (grams)	End-Pd-Load	WNS_Score
1032	1	Tippy Dam	5.34	4.08	4
1034	1	Tippy Dam	6.46	2.40	3
1036	1	Tippy Dam	5.97	4.19	2
1037	1	Tippy Dam	5.69	4.21	2
1038	1	Tippy Dam	5	4.79	3
1039	1	Tippy Dam	6.54	5.39	2
1040	1	Tippy Dam	6.34	4.72	1
1041	1	Tippy Dam	5.66	4.11	0
1043	1	Tippy Dam	5	4.37	1
1044	1	Tippy Dam	na	3.56	0
1045	1	Tippy Dam	6.3	4.84	2
1046	1	Tippy Dam	6.59	5.15	2
1047	1	Tippy Dam	4.39	4.94	2
1048	1	Tippy Dam	5.75	4.42	2
1065	1	Norway Mine	ND	4.06	2
1066	1	Norway Mine	6.01	3.76	1
1067	1	Norway Mine	6.07	4.00	1
1068	1	Norway Mine	4.74	3.88	2
1069	1	Norway Mine	7.45	3.51	2
1070	1	Norway Mine	6.37	3.98	2
1071	1	Norway Mine	5.71	3.67	1
1072	1	Norway Mine	6.1	4.02	1
1073	1	Norway Mine	5.8	4.28	1
1074	1	Norway Mine	5.75	3.47	2
1075	1	Norway Mine	5.95	4.29	2
1076	1	Norway Mine	5.37	3.99	0
1077	1	Norway Mine	6.32	4.41	2
1078	1	Norway Mine	4.82	3.11	1
1079	1	Norway Mine	5.1	3.54	1
1050	2	Tippy Dam	5.97	2.53	0
1051	2	Tippy Dam	6.36	2.57	1
1052	2	Tippy Dam	5.91	3.94	1
1053	2	Tippy Dam	6.52	4.04	0
1054	2	Tippy Dam	6.06	3.03	0
1055	2	Tippy Dam	6.9	2.41	0
1056	2	Tippy Dam	6.6	3.04	1
1058	2	Tippy Dam	6.44	3.52	0
1059	2	Tippy Dam	6.01	4.68	1
1060	2	Tippy Dam	4.81	4.38	2
1061	2	Tippy Dam	6.3	3.45	2
1062	2	Tippy Dam	5.19	2.88	0
1063	2	Tippy Dam	6.16	3.64	2
1064	2	Tippy Dam	5.87	4.04	2
1080	2	Norway Mine	4.28	3.92	1
1081	2	Norway Mine	5.54	4.04	2
1082	2	Norway Mine	ND	3.24	1
1083	2	Norway Mine	5.32	4.34	1
1084	2	Norway Mine	5.99	4.82	1

BatID	Chamber	Source	End weight (grams)	End-Pd-Load	WNS_Score
1085	2	Norway Mine	5.69	4.49	2
1086	2	Norway Mine	5.76	4.18	2
1087	2	Norway Mine	5	3.44	2
1088	2	Norway Mine	6.2	4.15	1
1089	2	Norway Mine	5.95	4.17	1
1091	2	Norway Mine	6.07	4.40	1
1092	2	Norway Mine	5.7	4.59	2
1093	2	Norway Mine	5.55	2.68	0
1094	2	Norway Mine	6.33	4.26	1

E) Density plots of initial and endpoint Pd loads between the two source locations

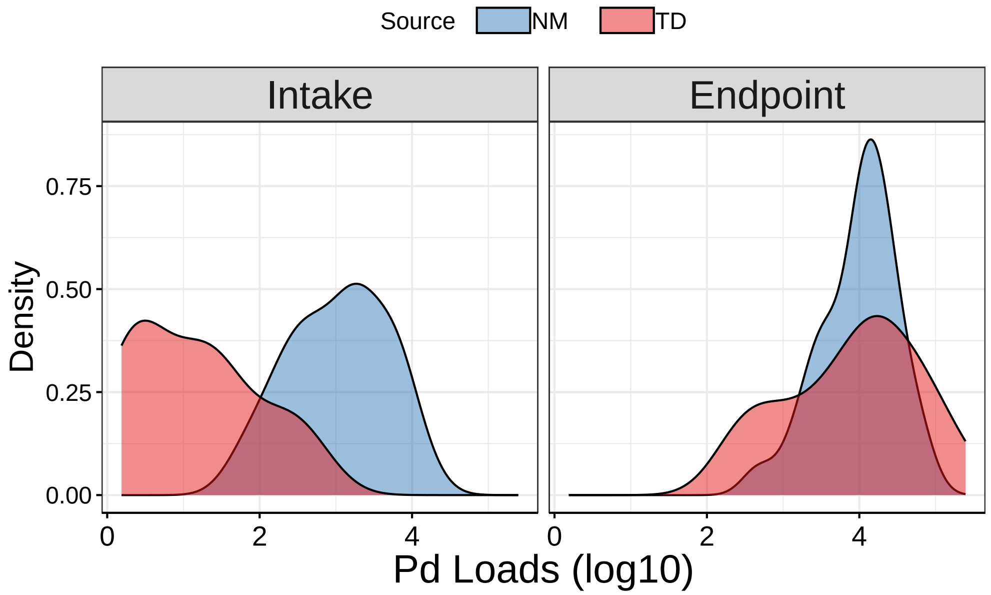

Figure 3: Density plot of log10 transformed Pseudogymnoascus destructans (Pd) loads of bats (Myotis lucifugus) used in the survival experiment. The plots show the weight distribution of bats from the two populations from hibernacula in Michigan (red for Tippy Dam (TD) and blue for Norway Mine (NM)) at intake (start of the experiment), and at endpoint (the end of the experiment).

F) Field Pd load analysis

On February 29th and March 1st 2025, we sampled 30 individual hibernating bats in hibernacula in Michigan, 30 bats in Tippy Dam and 30 bats in Norway Mine, respectively, for Pd load. We swabbed the length of the dorsal side of wing membranes three times per side for each bat with a pre-wetted, sterile cotton swab. The swabs were initial stored on ice in a cooler in the field and shipped to the U.S. Geological Survery, National Wildlife Health Center. The swabs were then stored in reagent grade sterile water at -20 °C, before quantifying Pd load through quantitative polymerase chain reaction (qPCR). After swabbing we identified sex of each individual. We used a linear model to test for difference in log10 transformed Pd load between the two source population. We included sex as an additional parameter. Our model indicated that bats from Tippy Dam had lower Pd loads compared to bats from Norway Mine. Sex of bat had no effect on Pd levels. These results further illustrate that Tippy Dam may have unique environmental characteristics that reduce Pd growth. However, future work would provide more information to test this hypothesis.

Table 3: Field Pd modeling results

Parameter	Est.Coeff	CI-2.5%	CI-97.5%
Intercept	3.10	2.79	3.41
Site:TippyDam	-0.39	-0.74	-0.04
Sex:Male	0.08	-0.26	0.43
SD	0.69	0.58	0.83

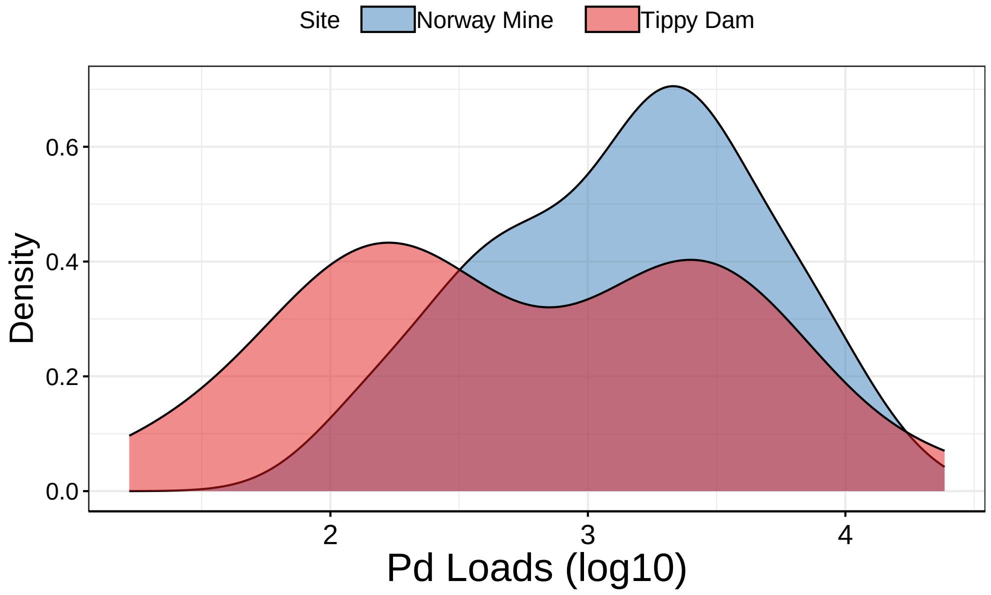

Figure 4: Density plot of log10 transformed Pseudogymnoascus destructans (Pd) loads of hibernating bats (Myotis lucifugus) collected in the field at both Tippy Dam (Red) and Norway Mine (Blue) hibernacula in Michigan
